# Biostimulant Substances for Sustainable Agriculture: Origin, Operating Mechanisms and Effects on Cucurbits, Leafy Greens, and Nightshade Vegetables Species

**DOI:** 10.3390/biom11081103

**Published:** 2021-07-27

**Authors:** Francesco Cristofano, Christophe El-Nakhel, Youssef Rouphael

**Affiliations:** Department of Agricultural Sciences, University of Naples Federico II, Via Università 100, 80055 Portici, Italy; francesco.cristofano@unina.it (F.C.); christophe.elnakhel@unina.it (C.E.-N.)

**Keywords:** horticulture, amino acids, fulvic matter, growth regulators, ROS, antioxidants, silicate, tomato, lettuce, cucumber

## Abstract

Climate change is a pressing matter of anthropogenic nature to which agriculture contributes by abusing production inputs such as inorganic fertilizers and fertigation water, thus degrading land and water sources. Moreover, as the increase in the demand of food in 2050 is estimated to be 25 to 70% more than what is currently produced today, a sustainable intensification of agriculture is needed. Biostimulant substances are products that the EU states work by promoting growth, resistance to plant abiotic stress, and increasing produce quality, and may be a valid strategy to enhance sustainable agricultural practice. Presented in this review is a comprehensive look at the scientific literature regarding the widely used and EU-sanctioned biostimulant substances categories of silicon, seaweed extracts, protein hydrolysates, and humic substances. Starting from their origin, the modulation of plants’ hormonal networks, physiology, and stress defense systems, their in vivo effects are discussed on some of the most prominent vegetable species of the popular plant groupings of cucurbits, leafy greens, and nightshades. The review concludes by identifying several research areas relevant to biostimulant substances to exploit and enhance the biostimulant action of these substances and signaling molecules in horticulture.

## 1. Introduction

Modern agriculture faces critical challenges that need to be addressed in order to feed the world’s population. It is estimated that there are currently over 7 billion people and, based on current trends, in 2050 the global population will reach 9.7 billion [1]. This increase will result in a rise in the demand for foodstuffs that is estimated to be 25 to 70% greater than what is currently produced [2]. Extreme weather events such as extreme heat and cold may be related to global warming-derived climate change [3], a phenomenon to which agriculture contributes by the way of emissions of greenhouse gases [4] and needs to be addressed in order to continue growing foodstuffs and also increasing production. To further add complexity to the matter, there is a necessity for agriculture to cut down on the use of resources, particularly on fertilizers, since the misuse of these chemicals has brought degradation of the land and eutrophication of the water; the misuse of low quality irrigation water coupled with intensive cropping practices has also brought widespread salinization of the soil [5]. It is estimated that 40% of the total arable land suffers from reduced fertility, and further expansion due to increased needs jeopardizes both plant and animal biodiversity [6]. The scenario described here poses the problem of increasing yields whilst reducing or minimizing the environmental impact.

The use of plant biostimulants (PBs) seems a valid strategy for the enhancement of sustainable practices. The latest regulatory framework in Europe defines PBs as products that should not be evaluated against their nutrient content; PB effects include increased plant nutrient absorption and use efficiency, tolerance to abiotic stress, and lastly, better produce quality [7]. Regulatory bodies in the US have yet to provide a formal definition; nevertheless, there is one pending approval that is sufficiently similar to what is effective in Europe [8].

Biostimulant substances (BSs), the subject of this work, are a diverse family of products that include silicon, seaweed extracts (SWEs), protein hydrolysates (PHs), and humic substances (HSs). The mechanisms of the biostimulant effects stem from a variety of factors, starting from the source materials and the production methods with which it becomes the final commercial product [9,10,11]. Product variety notwithstanding, at an abstract level, the stimulation of plant growth and productivity stem from the presence of active molecules such as peptides, algal polymers, and molecular structures that mimic and/or induce the production of phytohormones [11,12], stress-averting antioxidants [13,14,15], and plant growth regulators [12,16,17]. By modulating the plants’ primary and secondary metabolism, PBs elicit a cascade of messages that result in the in vivo results seen in the available literature, which reflect the claims imposed by the European Union for the category [18].

The outlook on the products is generally favorable based on the available literature; however, results are subject to combinatory effects due to the interaction with biostimulant management and environment. The former is due to the application mode criteria that the biostimulant user employs, based upon the mode of application (foliar application, substrate drench, seed coating), dosage regimen, frequency, application timing, and lastly, growing conditions. In their recent review on biostimulant application on fruit trees, Basile and collaborators [19] speculated that the biostimulant effect may not always be consistently efficacious compared to greenhouse-grown plants. The same authors attributed the elevated efficacy in the latter group to a higher application frequency and to the controlled growing environment, which may improve biostimulant uptake, especially when applied via foliar sprays; in particular, this mode of application greatly benefits from high humidity (which can be managed in controlled environments) and leaf porosity, which is a species-dependent characteristic [20].

With these factors in mind, we aimed to collect and sift through the currently available literature on the effects that BSs have on the most widely cultivated species of the horticultural groups of cucurbits, leafy vegetables, and nightshades or solanaceous plants. Other than the familiarity most people have with these groupings, and the clear economic importance of belonging crops such as tomato (*Solanum lycopersicum* L.), lettuce (*Lactuca sativa* L.) and cucumber (*Cucumis sativus* L.), the large availability of literature on biostimulant applications make them prime candidates for evaluation. In particular, the consulted studies vary in cultivation environment from open field, to greenhouse, to growth chamber, cultivation systems such as soil-based, soilless, and hydroponics, coupled with the plethora of BSs used. To provide an all-encompassing understanding of the mode of actions and/or of the effects these substances have on these important crops, we evaluated the origin, mode of action and the operating mechanisms through which these products modulate plant physiology, and their effects on the wide variety of case studies found in the literature. We tackled the ameliorative effects on abiotic stresses, the increase in plant growth, yield, and product quality attributes. We also envisioned the direction biostimulant science may embody in the future, and where research efforts need to be put forth.

## 2. Origin, Composition, and Mode of Action of Biostimulant Substances

To garner a better understanding of the biostimulant substances found in this review, we have proceeded throughout the body of the work to divide them in silicon, SWEs, PHs, and HSs. The rationale for this order stems from the starting consideration that out of all the considered substances, silicon is the only inorganic biostimulant currently present, and, therefore, should be described first. Furthermore, we have proceeded to divide the organic-derived biostimulants on a market prominence basis, as research shows that SWEs, the most commonly used product category, represent 37% of all the PBs market share, with PHs and HSs following suit, with a combined 50% [21].

### 2.1. Silicon

Silicon is the second most abundant element in the Earth’s crust, and while it is universally not considered as an essential nutrient for plant growth, it is proven to have biostimulant action [22]. Sources of this material for use in agriculture include wollastonite (CaSiO_3_), residues of blast furnaces, and usually, rice-derived straw [23]. Again, though not essential for most plants per se, silicon in the growing medium still provides clear advantages to the grower, such as the mechanical strengthening of tissues that prevents lodging, increases in fruit firmness [24], and also favors the formation of physical barriers to help fend off fungal [25] and insect [26] attacks.

Plant species can be grouped based on their absorption of silicon from the growing medium as high, intermediate accumulators, and excluders. The rationale for this grouping is that depending on the species affinity for the element, absorption may be more, equal or less than what enters through water uptake only; this can be seen by comparing the percent ratio of silicon in dry weights, which is the highest in accumulators [27]. Root absorption of silicon from the nutrient solution is usually made possible by aquaporin-type channels, in particular nodulin 26-like intrinsic proteins (NIPs) [28]. Interestingly, due to similar structures, arsenite is also transported via the same Si channels [29]. In theory, administering silicon to arsenite-containing mediums could actually alleviate the negative physiological effects of arsenite by way of dilution only.

The literature on the use of silicon is well-centered on its use as a stress alleviator, and a recent review by Zhu and collaborators [30] highlighted its role in regulating ion homeostasis, modulating water balance, and the activity of antioxidant molecules.

Ion homeostasis is fundamental to guarantee adequate growth. A high concentration of salt causes protein and membrane destabilization and also ion imbalances, because Na^+^ at high concentrations competes for the same high affinity K^+^ transporter, hence reducing potassium availability [31,32]. Heavy metals such as Al, Mn, and Cu, which were encountered in the available literature, compete with essential elements such as Ca and Mg and, by substituting themselves to the latter ones, disrupt essential reactions [33].

The internal mechanism that plants use to counteract the effects of salt stress is by the exclusion of the dangerous Na^+^ by expelling it in the apoplast or by moving it to vacuoles. This is conducted by Na^+^/H^+^ antiporters of the SOS and NHX type in the plasma membrane and vacuoles, which, when located on the root, can directly expel the toxic ions from the plant [32]. In order for these proteins to function, there is an elevated need for H^+^ ions to be expelled from the cytosol to form the electro-chemical gradient, which in turn creates the electromotive force that moves sodium away; this is performed by the way of H^+^-ATPases. Whilst evidence is still uncertain, or at least it seems species-dependent on the role of silicon as a SOS modulator [34,35], its influence has been proven on the upregulation of H^+^-ATPases [36], even on a plant of horticultural interest such as tomato [37], where LHA1 and LHA2 proteins were upregulated after silicon amendment, and cucumber, where it promoted the expression of vacuolar Na^+^/H^+^ exchanger gene NHX1 [38]. In the tomato case, since the plants were grown in a high pH environment (9), it could be inferred that Si could be used to ameliorate pH stress by lowering the rhizosphere pH via the excretion of protons, thus augmenting abiotic stress resistance [37].

Silicon is also used by plants to augment their defenses against the entrance of toxic ions via the root apoplast. Sodium ions manage to cross the symplast through the apoplastic pathway by way of what is called a bypass flow [32]. A bypass flow is formed where the apoplastic barriers, i.e., Casparian bands and suberin lamellae, are not completely developed. Silicon supplementation has been shown to promote the growth of those barriers in some species, including Onion (*Allium cepa* L.), a Si-excluding species of horticultural value [35], thus reducing the bypass flow.

Additionally, related to the apoplast is the mechanisms with which silicon alleviates ion toxicity. The formation of hydroxy-aluminum silicates in the apoplast of the root apex may be the reason for the reduction in apoplastic Al mobility [39], and the binding of excess Mn and Cu to cell walls in cucumber plants [40,41] may explain the increased resistance to an excess of these elements.

Improving water balance is also a way with which silicon alleviates salinity and drought stresses, since the two are alike, as excess soil-borne salt contents increases the osmotic potential of the circulating solution, thus generating a water deficit [42]. As stated before, silicon transport is mediated via AQP-like proteins, which also facilitates cell water intake. The upregulation of silicon-transporting aquaporins as seen in cucumber plants [43], which also translated in higher conductance, may also explain the effect on salinized tomato plants that showed higher water contents compared to the controls [44] and on pepper plants showing an enhanced leaf water potential [45]. The benefits of silicon on plant water balance may also come from the increased amounts of osmoprotectants such as proline and glycine betaine as seen on pepper, cucumber, and tomato plants [46,47,48].

At last, further explanation on the inner workings of silicon in regard to abiotic stresses is found in ROS response and antioxidant modulation. ROS levels are usually kept in balance by antioxidant molecules, but stressful events can induce plants to produce toxic levels of these molecules that prove costly for growth and yield [49]. Since ROS homeostasis drives organ growth, in particular root growth, and favors germination [50,51], it is then of crucial importance to maintain it. Silicon acts by modulating antioxidant activity and, in particular, improves on the production of ROS scavenging enzymes such as superoxide dismutase (SOD), ascorbate peroxidase (APX), catalase (CAT), and glutathione peroxidase (GPX) [13,37,52].

As the works included in this review seem to indicate, there is no strong body of evidence suggesting an increase in plant growth, performance, and/or product quality in non-stress situations. Silicon as a biostimulant research seems to be well focused on the amelioration of stresses because, as shown, there is sufficient corroborating evidence in that direction. Regardless, from all the information we have thus far gathered on silicon and its effect on stress control, we can conclude that the use in horticulture seems to be beneficial without any particular side-effects, though there could be a non-defined dose ceiling above which treatments could prove to be detrimental. Contrary to other products, silicon usage is quite easy to recommend since—aside from specific formulations such as silicon nanopowder—it can be easily and conveniently added to nutrient solutions and fertilization regimens by the way of orthosilicic acid and/or silicate salts. Still, as silicon absorption varies on a species-to-species basis, providing a single one size fits all dosage could prove difficult and this could be a new research area, maybe integrating mixtures of silicon and other BSs with an agonistic molecular approach.

### 2.2. Seaweed Extracts

SWEs are products usually deriving from the water/solvent extraction and hydrolysis from algae biomass of the genera *Ascophyllum*, *Ecklonia*, *Macrocystis*, and *Durvillea* [53], though more are currently under investigation. Production methods are not standardized and often proprietary: in a 2019 review on *Ascophyllum nodosum* extracts, it was found that eight modes of extraction are currently being employed today [11].

As such, SWEs pose some risks in discussing them as a single entity due to their wildly varied selection of species, the inherent variation of their constituents based on climate and season, and the plethora of extraction modes [11]. Further increasing variation is the inclusion of either multi-species products, such as in the case of ‘TAM’, a mixture of *Ulva lactuca*, *Jania rubens*, and *Pterocladia capillacea* extracts [54], or non-algal derived matter such as ‘Amalgerol’, a mixture of oils and *Ascophyllum nodosum* extracts [55]. In the aptly named paper ‘Comparative Transcriptome Analysis of Two *Ascophyllum nodosum* extract biostimulants: Same Seaweed but Different’, Goñi and collaborators [56] showed that two *A. nodosum* extraction methods yield wildly different commercial products, which, in turn, provide for significantly different results in both formulate composition and plant response. To further prove this point, in studies on either lettuce and tomato plants where two *Ascophyllum nodosum* that varied on extraction temperature were tested, Guinan, Dell’Aversana, and their collaborators [57,58] proved that the extracts performed differently in ameliorating salt stress.

Nevertheless, there are some fundamental qualities related to seaweed extracts that can be indicated as responsible for the biostimulant activity and may be shared by the majority of the products. In 2010, when defining the active molecules in SWEs, Cragie [59] divided them into plant hormones such as abscisic acid (ABA), gibberellic acids (GAs), auxins, brassinosteroids [60], and cytokinins (CKs) [11,60,61], growth regulators such as betaines [62] and algal polymers, especially polysaccharides such as alginates, fucoidans, mannitol, and laminarin [11]. Eckol, a bioactive molecule extracted from the seaweed *Ecklonia maxima*, which incidentally has been widely used in a variety of research covered by this work, has shown both an auxin-like and a general growth promoting effect in spinach plants [63]. Furthermore, when evaluating the biostimulant activity of five commercial products derived from the genera *Laminaria* and *Ascophyllum*, Ertani and collaborators [61] found that they variably increased root system growth and plant nutrition. In particular, they found that one of the tested *Ascophyllum nodosum* had higher contents of auxin and cytokinin, which they found to be responsible for the increased lateral root hair production. Other corroborating evidence of hormone-like effects is found in the enhanced expression of flowering genes in tomato plants [64].

As thus, the hypothesized mechanism of increased plant growth and yield seen in the works detailed here, seem to result from the cascade of signals stemming from the application of the products. By regulating the plants’ phytohormone signaling, SWEs can improve nitrogen, carbon metabolism, and the acquisition of important nutrients that result in better physiological states and, thus, better growth [16]. Moreover, as ABA and CKs are of crucial importance in the case of abiotic stresses, they are also related to the quality improvements denoted here. ABA-mediated signaling is linked with the induction of enzymatic and non-enzymatic antioxidant systems [65], which include phenolics, flavonoids, and ascorbic acid that benefit human health [66] and has been seen in the variety of the species covered by this work.

Furthermore, the stress-related biosynthesis of ABA is one of the fastest ways plants respond to unfavorable conditions: an accumulation of ABA reduces water loss by stomata closure, which is crucial in the case of drought and salinity stresses. CKs, having ABA antagonistic effects, may, in some cases, further enhance stress resistance by partially inhibiting ABA accumulation [67]. SWEs rich in ABA-like molecules have proven to inhibit germination and root growth in Arabidopsis, which were reverted when tested on an ABA insensitive mutant [68]. These results are in agreement with a previous 2013 study [69] on *A. nodosum* extracts, though the concentration of hormones in the extracts were deemed so low that it was speculated that the effects came from hormone production inducing molecules.

Brassinosteroids are a class of phytohormones—or PGRs, depending on the source—that are found in many plant tissues and are needed for plant development and response to stresses. The two known active brassinosteroids, brassinolide (BL) and castasterone (CS), were both found in the commonly used seaweed formulation ‘Kelpak’ [60]. The mechanisms of the brassinosteroid-mediated amelioration of stresses are still to be completely elucidated, due to the interactions with other factors such as GAs and salicylic acid. However, there is a sufficient amount of evidence pointing to a brassinosteroid-induced modulation of antioxidant activity and a subsequent reduction in oxidative stress induced by drought, salinity, extreme temperatures, and flooding, which was described in a 2015 review by Vardhini and Anjum [70].

Alginate-derived oligosaccharides induced a drought tolerance in tomato plants that determined a higher biomass, lower MDA contents, and a higher proline content and antioxidant activity (SOD and Peroxydase or POD) [14,71].

With the regard of abiotic stresses, we found ourselves agreeing with the hypothesis put forth by Van Oosten and collaborators [72], whereby SWEs with their application modulates ROS scavenging mechanisms—thus reducing oxidative stresses—reduce ion toxicity by modulating the ABA and CK pathways, therefore, improving membrane stability, and lastly, promoting osmoprotection by increasing the contents of compatible solutes such as proline and providing plants with betaines. By extension, through these mechanisms, SWEs ameliorate stresses in the critical phases of plant biology, which results in an increased plant growth, yield, and antioxidant activity.

A new frontier for SWEs could be as molecular priming agents not unlike what is described by Kerchev and collaborators, which hypothesized that activating phytohormone signaling and antioxidant systems results in better protection when a later stress is applied [73]. Proof of this theory can be found in the results obtained in a recent tomato study [74], where primed tomato seed produced plants showing strong growth and yield when grown in a saline environment. Nonetheless, care should still be taken because, as we emphasized before, the available commercial products are so different in their composition, where providing a catch-all explanation of their workings becomes very hard. A completely new, ‘just what works’ molecular approach may be needed in order to create standardized products that satisfy the needs of a market that will become more demanding as foodstuff needs become greater and abiotic stresses more common.

### 2.3. Protein Hydrolysates

PHs are commonly defined in the literature as ‘mixtures of polypeptides, oligopeptides and amino acids that are manufactured from protein sources, using partial hydrolysis’ [75]. Hydrolysis systems include chemical, either acid or basic, and enzymatic by way of proteolysis [9].

The source materials include various protein matrices, which include animal-derived epithelium and connective tissue, plant-derived biomass such as alfalfa and soybean, and algal protein [9]. The interaction between the extraction method and the source material induces a variation among products, which differ by various parameters, such as the free amino acid to protein/peptide rate, amino acid chirality, molecular weight of the constituents and electrical conductivity. When Cavani and collaborators [76] tested 22 different products, they found total amino acidic contents to vary from 5.3 to 52.5%, free amino acidic contents from 0.76 to 19.6%, and electrical conductivity from 3.9 to 20.0 dS m^−1^. In particular, products stemming from the chemical hydrolysis of the source matrix by way of strong bases or acids under a high temperature and pressure, can lead to products with high free-amino acid contents, racemization, and high electrical conductivity. All of these factors may add up to products, which may prove to cause phytotoxicity symptoms, especially in the case of a high dosage and number of administrations [76,77].

The uptake of PHs biostimulants happens through either foliar or root absorption, and the absorbed peptides and amino acids can be readily transformed in whichever compounds plants need. However, product uptake depends on application, modality, and environmental factors. Substrate application may result in plants taking up only around 6–25% amino acids due to microbial competition [77], whilst foliar uptake is mediated by wind speed, humidity levels, stomata opening and number, and leaf cuticle thickness [20].

As PHs are nitrogen-rich products, the effects on plant growth could very well stem from nitrogen fertilization alone. Whilst large-scale biomass hydrolyzation could be employed in the future to reduce waste, current biostimulant application rates consist of 1–3 L of commercial formulation per hectare of soil, with products themselves having nitrogen contents of 4 to 8% [76]; such figures show that the biostimulation seen in the literature does not depend on nitrogen fertilization alone. As Colla and collaborators [12] summed up in 2017, the mechanisms behind the plant physiological response due to PHs’ biostimulant application can be summarized as the increase in root growth due to the hormone-like activities, increased nutrient uptake, the stimulation of carbon and nitrogen metabolism, and the modulation of the antioxidant systems.

The most plausible explanation to the increased root growth is to be found in the presence of signaling peptides, which act as plant growth regulators (PGRs). One such peptide is the root hair promoting peptide, which has been found in a commercially available product [78]. It is probably due to the presence of those molecules that auxin-like activities were multiple studies on either vegetal and animal-derived PHs in both stress and non-stress conditions [15,79], and to further corroborate this hypothesis, new research found out that by molecular fractionation of commercially available products, it possible to obtain specific formulations that could be comparable in efficacy to synthetic auxin in root growth induction [80]. Furthermore, in stressful conditions such as low nutrient availability, protein hydrolysate-dependent root growth may also come from the modulation of salicylic acid production, which may, in turn, induce lateral root growth [81]. However, and understandably, due to dissimilarities in source materials both the increases in root number and growth have proven to show a degree of variance, even when extraction methodology is consistent [82].

Carbon and nitrogen metabolism stimulation by PHs biostimulants is attributed to an increase in the activity of enzymes in the tricarboxylic acid cycle (TCA), and the increase in enzymatic activity in the nitrogen metabolism and uptake, due to the up-regulation of transcript levels related to nitrogen transporters [83].

The modulation of plants’ antioxidative systems is probably due to an enhanced cell-to-cell message transduction after the application of the products. In a 2019 metabolomic study on tomato plants, Paul and collaborators [84] found that the plant response to biostimulant application revolves around the ROS plant signaling network. Among their findings, treated plants showed increased contents of antioxidant compounds such as phenolics and carotenoids. An increased antioxidant molecule content is particularly favorable from a product quality standpoint, and it has been found throughout the consulted literature.

The evidence surrounding the pathways with which PHs work to ameliorate abiotic stress, relates to both product composition and the induction of plants’ osmotic regulation and antioxidative systems. PHs contain osmo-regulating molecules to ameliorate drought and salt stresses; some products contain significant quantities of plant compatible osmolytes such as proline, the concentration of which depends on the extraction methodology and protein source—animal or vegetal [85,86]. PHs-mediated plant osmoregulation may also work by the way of eliciting the production of osmolytes such as trehalose that was found upregulated in tomato plants [87]. The second pathway to a better stress resistance is probably due to an enhanced message transduction. Phytohormone and ROS signaling-mediated messages may favor the production of stress-averting antioxidant molecules such as ascorbic acid, tocopherols, and antioxidant enzyme activities, an increase that has been found in several studies [15,86,88], and may further boost the nutraceutical quality of the products.

In conclusion, signaling peptides research may be the future of a particular product category, and since the key to PH biostimulants may very probably lie in these molecules, it would be interesting to see what further research may yield in terms of better performance of the products, and if a different formulation could be made according to satisfy individual needs.

### 2.4. Humic Substances

HSs described as dark colored, heterogenous aggregates of organic matter, are the result of micro-biotic metabolism, extremely resistant to degradation, and one of the most abundant organic materials on the planet [89]. A traditional—though nowadays criticized—subdivision of this material splits it into the following three groups: humin, the non-water-soluble portion; humic acids (or HAs), soluble in pH > 2 media; fulvic acids (also FAs), which are water soluble [90]. This division, as obsolete as it is, is the most used in all the consulted literature, as it provides a way to produce a meaningful distinction between products, i.e., a humic acid-based product is different than a fulvic acid-based one.

Nevertheless, as Muscolo and collaborators [10] explained in their 2014 article, HSs are now recognized as the structural association of mixtures of small and distinct organic molecules, which are linked together via hydrogen bonds and hydrophobic interactions, and that their diversity is due to different external perturbations and resource usage strategies employed by the ecosystems. This definition suggests that, rather than the molecular constituents, it is molecular structure and size that seem to be critical in plant–HS interaction. Due to the presence of a high number of oxygenated functional groups (CO_2_H_2_, OH phenols, C=O) [91], HSs in the growing media improve plant nutrition by forming complex, stable bonds with micro and macronutrients [17]. While this effect may vary based on the source material, genesis, application dose, and characteristics of the growing medium, it generally results in elevated macro and micronutrient absorption by plants, which may at least partly explain the growth results clearly seen in tomato, pepper, and cucumber plants [92,93,94,95].

Indol-3-acetic acid (IAA) and CK content in HS may also explain the improved growth and yield parameters seen in this review. As expected, CK content seems to depend on source material [96] and, likewise, IAA-like activity, but can still be substantial enough to rival the results obtained with synthetic IAA [97], and, thus, elicit increases in root growth [98]. What also may have come into play is the stimulation of root plasma membrane H^+^ ATPase by auxin-like compounds or nitric oxide-dependent pathways [99], which may create an electrochemical gradient that could facilitate ion uptake [100]. Enhanced nutrient uptake clearly shows an interesting use case for the amelioration of stresses due to alkaline soils, where micronutrients such as Fe are unavailable to plants; water extractable humic fractions have proven to be successful in enhancing the Fe nutrition in tomato plants, even at an elevated soil pH [101], and assisting Fe-deficient cucumber plants in acquiring Fe more efficiently than other organic ligands [102].

What most likely gives the ability of HSs to alleviate abiotic stresses is the interaction with plant roots. While in optimal conditions HSs induce the production of ROS in plants to the point of which excessive doses may actually be detrimental to plant growth [49], it seems that under high stress conditions, they balance excessive ROS response by modulating antioxidant enzymes such as SOD, APX, and POD and determine increases in osmolites such as proline [103,104,105]. As Garcia and collaborators [99] summarized, the effect of HSs on plant development due to their structure may depend on the induction of signaling networks composed of phytohormones and messengers such as ROS and Ca^2+^. As with SWEs and PHs, ROS-mediated messages may, in turn, favor the production of human-benefitting phytochemicals, and the evidence points to this being the case in most of the studied species in this work.

In their review, Shah and collaborators [17] called HSs plant tonic for the multitude of effects they have on plant growth and development and advocated for research of the mechanisms that govern HSs-induced effects. We also express the need to further explore HSs’ use in stressed horticultural plants, as they represent widely cultivated and often lucrative cash crops. Widespread research in this area may also mean the future widespread adoption of HSs, as knowledge regarding the molecular and biochemical pathways through which they work may standardize results, thus favoring the adoption of underutilized organic waste for humification, which would prove an environmentally friendly use of resources.

## 3. Cucurbits (*Cucurbitaceae*)

### 3.1. Biostimulants Substances to Increase Cucurbit Resilience to Stress

Cucumber (*Cucumis sativus* L.), the model cucurbit and a Si accumulating species [106], has shown to be responsive to silicon treatments. An addition to the nutrient solution of either sodium silicate (Na_2_SiO_3_), sodium silicate-derived metasilicic acid (H_2_SiO_3_), or engineered nanosilica at the rate of 0.3 [43,107], 0.8 mM [104], and 200 ppm [108], respectively, have proven to significantly increase the germination rates, fresh and dry weights, decrease the sodium content in roots or leaves, and increase the root hydraulic conductivity of salinized cucumber plants. Moreover, a better physiological status, as in higher photosynthetic rate and F_v_/F_m_, was recorded than the untreated salt-stressed controls, and comparable results were also obtained when combined heat and salinity stresses were applied [109,110,111].

This is also true for zucchini squash (*Cucurbita pepo* L.) and watermelon (*Citrullus lanatus* Thunb.), where the application of potassium silicate (K₂O₃Si) and silicic acid at the rate of 1 mM and 4 mM silicon, respectively, via the nutrient solution to greenhouse grown-plants has proven to improve the condition of the stressed controls such as the lower net photosynthesis and fruit yield [52,112], and also reduced the fruit weight loss during storage, though not to an extent that increased market life [113].

In water-stressed cucumber plants, silicon treatments exhibited higher leaf area, fresh and dry biomass, antioxidant activity, and yield [114,115,116].

Iron deficient [117,118,119] and micronutrient-deprived [119] cucumber plants also benefitted from silicon supplementation, which prevented Fe deficiency symptoms [118,120], an effect that was more evident at a higher pH (6.0 vs. 5.0) [117]. Moreover, silicon partly ameliorated zinc deficiency symptoms [119]. Furthermore, silicon has also shown a protective effect against the excess concentration of ions, particularly aluminum [120], manganese [41,121,122,123], and copper [40,124].

Lastly, silicon was found to be effective against the symptoms of cucumber autotoxicity, an intraspecific allelopathy that limits germination rates, seed vigor, and root growth [123,125].

We were able to find evidence on the effects of SWEs based on one article by Rouphael et al. [126] on greenhouse-grown zucchini squash plants subjected to three salinity levels (20, 40, and 60 mM). When averaged over salt treatments, the five, bi-weekly foliar applications of the commercial Ecklonia maxima extract ‘Kelpak’ at the rate of 3 mL L^−1^ improved marketable yield and shoot dry biomass by 12 and 17.4%, respectively, compared to the untreated controls. Moreover, salinized plants produced better quality fruits, as expressed by the TSS content and darker color.

Fe-deprived (10% of the full strength nutrient solution, or 4 μmol) cucumber plants grown in a growth chamber and subjected to two weekly treatments of the ‘Trainer’ PHs at the rate of 3 mL L^−1^ showed double the shoot iron contents when compared to the untreated controls. Moreover, whilst the Fe-derived controls showed a 30% reduction in the relative chlorophyll content compared to the full-strength solution, biostimulant-treated plants only showed a 12% reduction [127].

Evidence of the effect of HSs on stressed cucurbits is scarce. In a 1999 study by Demir and collaborators [128], HA was applied to cucumbers grown in soil supplemented with 28 and 56 mM of sodium chloride Kg^−1^ soil. The plants treated with HA showed a higher yield compared to the non-treated ones, though the exact figures were not published.

Table 1 shows an overview of the effects biostimulant substances have on stressed cucurbit crops.

### 3.2. Implication of Biostimulant Substance Treatments on Cucurbit Growth and Yield

Literature in favor of a role of silicon as a growth-improving substance for cucurbits seems to be lacking. In a 2018 greenhouse cucumber study [129] on silicon treatments, either via the soil incorporation of affordable *wollastonite* or via irrigation with soluble potassium silicate, did not result in significant increases in either growth or yield. Silicon treatments also do not seem to improve cucumber nutritional status in leaf tissues as no significant differences were noted between the micronutrient and macronutrient contents of treated and untreated plants [130].

Conversely, an increase in per-plant yield of 17.3% was recorded in silicon-treated watermelon plants in the second growing season of a two-year experiment (2005 and 2006), which may suggest species-dependent effects [131].

Ten-percent foliar sprays of seaweed extract from the species *Macrocystis pyrifera*, *Grammatophora* spp., *Bryothamnion triquetrum*, *Ascophyllum nodosum*, and *Macrocystis integrifolia*, the first two laboratory made and the latter being commercial products ‘FulvimaxAT’, ‘SeaplantAT’, and ‘GaiaAT’, respectively, were tested on greenhouse cucumber grown in sand and vermicompost against a control irrigated with Steiner solution, a standardized nutrient solution employed in agriculture. While the SWEs-treated plants showed a lower fruit size and weight compared to the nutrient-solution irrigated control, the *Bryothamnion triquetrum* treatments only showed a 7% reduction in fruit weight and an 8.3% reduction in yield [132].

Similarly, Hassan and collaborators [133] exchanged 25, 50, and 75% mineral fertilization of greenhouse-grown cucumber with bi-weekly foliar sprays at the rate of 2.5, 3.5, and 5 mL L^−1^ of ‘TAM’, an extract derived from *Ulva lactuca*, *Jania rubens*, and *Pterocladia capillacea*. Researchers found that ‘TAM’ successfully managed to produce a 51.9% average increase in yield compared to the normally fertilized control. These results, probably due to elevated nutrient use efficiency, show a possible usage of SWEs in reducing the fertilizer input.

HSs testing on cucumber plants goes a long way, as we found a 1981 paper [93] in which varying concentrations of FAs from 20 to 2000 ppm were administered to growth chamber-grown cucumber plants in addition to a Hoagland solution. The study showed significantly improved physiological parameters such as shoot height and length, leaf and flower number, and also enhanced nutrient (phosphorous, potassium, calcium, magnesium, copper, iron, zinc) concentrations in shoots.

Humic acid trials on cucumber plants in both growth chamber and greenhouse conditions also yielded similar results, with plants showing significantly higher dry weights compared to the untreated controls, suggesting higher nutrient absorption [92,134].

Moreover, greenhouse-grown cucumber treated with HAs via either foliar spray or soil applications at the respective rate of 20 and 30 mL L^−1^ recorded higher fruit yields of 14.9 and 14.5% [135]. Ekinci and collaborators, who tested varying dosage rates of HA soil supplementation (0.5, 1, 3, and 5 g kg^−1^ substrate) found that the addition of 3 g kg^−1^ of a substrate of calcium salts of the commercial HA formulate ‘Actosol’ provided 28.7% yield increases compared to the untreated control, and 14.4% compared to ‘Actosol’ alone [134].

Nevertheless, the literature shows that high dosages of HSs may actually be detrimental to the growth of cucurbits. Rauthan and Schnitzer [93] found that an over 300 ppm concentration of nutrient solution-dissolved FAs had proven detrimental to the effectiveness of the treatment, and similar effects were later denoted when cucumber seedlings were grown in greenhouse conditions in potting mix amended with varying concentrations of humates deriving from food waste and pig manure vermi-composts [136]. Whilst food-waste-derived HS was effective at increasing the shoot and root dry weights (28.6 and 18.5%, respectively) compared to the untreated controls at the lower dosage of 50 ppm, the latter pig-derived compost, other than being ineffective at lower dosages, i.e., less than 500 ppm, induced a reduction in the same parameters when applications were higher than 500 ppm, thus showing a variation between humate sources.

Further evidence is found in a watermelon study, where seedlings grown in a shade house and sprayed with various concentrations (0.4, 0.8, 1.19, and 1.59 mL of formulate per seedling) of commercial product ‘Humitec’ showed higher-than-control growth at the first three dosage regimens that regressed when the dosage was increased [137].

Table 1 shows an overview of the growth and yield-promoting effects biostimulant substances have recorded on cucurbit plants.

### 3.3. Cucurbit Fruit Quality Modulation after Biostimulant Applications

From the limited available literature, silicon does not seem to increase quality parameters such as total soluble solid and ascorbic acid content in greenhouse-grown zucchini squash, cucumber, and watermelon plants [113,129,131]. The visual aspect of the cucumber and watermelon fruits such as peel and pulp color also did not show significant differences following silicon treatments in either study [129,131].

Increased quality parameters in cucumber plants treated with HAs were found in two studies, which tested dosage rates and modes i.e., foliar and soil applications. In a 2011 greenhouse experiment [135], it was found that both applications modes increased fruit firmness, with the highest increment of +17.2% recorded by the 20 mL HA L^−1^ treatment group, when compared to the untreated control.

Nevertheless, the authors found out that foliar treatments might be the best use-case for improving cucumber fruit quality, as a 10 mL L^−1^ foliar regimen yielded the highest increments of total soluble sugars (+14.4%) and reducing sugars (+25.3%). In a later study by the same authors, a further increase to 20 mL L^−1^ foliar applications recorded the highest increases in fruit antioxidant activity, either lipophilic (+31.7%) and hydrophilic (+148%), total carotenoids (+74.2%), lycopene (+120.8%), and β-carotene (+92.8%) contents compared to the untreated controls [138].

Table 1 shows the cucurbit product quality modulation after biostimulant applications.

## 4. Leafy Vegetables

### 4.1. Biostimulants Substances to Increase Leafy Green Resilience to Stress

Lettuce (*Lactuca sativa* L.) seeds treated with 6 mM sodium silicate, in accordance with the results on other species, has proven to either improve or bring seed germination parameters to satisfactory levels when seeds were exposed to saline environments as high as 200 mM NaCl [139]. Moreover, when Milne and collaborators [140] evaluated greenhouse-grown lettuce plants subjected to nutrient solution to which 60 mM NaCl was added, they found that a 2 mM silicon treatment increased shoot and root fresh weights by 71.5 and 75.2%.

In greenhouse spinach (*Spinacia oleracea* L.) plants, the application of 50 mM NaCl kg^−1^ of soil and boron at the rate of 50 mg H_3_BO_3_ kg^−1^ resulted in higher fresh weights (+16.7 and +19.9%) compared to the unstressed control [47], which could be explained as the species is fairly tolerant to salinity, up to a soil salinity equivalent level of 4.5 dS m^−1^ [141]. Even still, a silicon supply at the rate of 2 mmol silicon kg^−1^ of soil resulted in higher fresh weights, in addition to an improved antioxidant activity compared to both non-saline control, boron, and saline treatments [142].

Further proof of the amelioration of boron toxicity by silicon treatments is found in a study by Gunes and collaborators [143], where silicon improved root dry weights and reduced the severity of leaf symptoms compared to the boron-stressed controls. Silicon-grown plants also showed lower malondialdehyde (MDA) and proline contents, suggesting lower oxidative damage and better osmotic balance [142,143].

Arsenic toxicity in lettuce due to arsenite and arsenate, paired with silicon administration was also evaluated in a 2015 growth chamber study [144]. Nutrient solution applications of arsenite and arsenate on lettuce over 0.1 μmol resulted in a decrease in fresh weight, in particular with the former treatment. Nutrient solution treatments at the rate of 1 mM potassium silicate decreased the effects of the arsenic-containing compounds and, in particular, across arsenate and arsenite treatments, increased the plant dry and fresh weights by 7 and 21% and by 5 and 14% for arsenate and arsenite, respectively.

The *Ascophyllum nodosum-*based, commercial SWE ‘Improver’ at the 0.3% rate improved the germination rates of heat-stressed (30 °C) spinach seeds by 25%, compared to the control. Moreover, seed priming with the biostimulant resulted in seedlings with a lower hydrogen peroxide and a decreased MDA content, suggesting lower oxidative damage [145]. Salt stress protection by SWEs was tested by using two commercial *A. nodosum* extracts obtained using two different extraction methods. Greenhouse-grown lettuce plants subjected to 80 mM NaCl stress were treated with an addition to the nutrient solution of varying concentrations of high (>125 °C, ‘Super Fifty’) and low (<75 °C, ‘Ecolicitor’) temperature extracts. ‘Super Fifty’ treatments proved to be the best performing by expressing comparable fresh weight numbers to the non-stressed controls, and a 42.53% increase when compared to the saline-stressed control, when tested at the lowest rate of 0.4 mL biostimulant L^−1^ nutrient solution. Furthermore, by determining the antioxidant activity of the two products, researchers found a 32-fold difference in favor of the high temperature extract, suggesting that the extraction method has a role in determining the extract properties [57].

The effect of a *A. nodosum* extract on potassium deficiency symptoms was also tested on greenhouse-grown lettuce. The foliar application of a solution containing 1% of the extract on potassium-deficient plants resulted in improved growth parameters that were comparable to the non-stressed controls. Moreover, treated plants showed higher photosynthetic activity, even when compared to the non-stressed controls and lower leaf fluorescence (F_v_/F_m_), thereby indicating a better physiological state [146].

Drought-stressed spinach plants grown in a growth chamber and treated with *A. nodosum* extract ‘Stimplex’ with various application modes (0.5% solution foliar, 50 mL of 0.5% drench, and combined applications) showed significantly higher leaf fresh and dry weights than the control treatment, with both foliar and drench being equally effective. The physiological parameters such as net photosynthetic rate, stomatal conductance, and transpiration were also increased by all treatments by 25, 71, and 42%, respectively [147]. Evidence of the effect of PHs on stressed leafy vegetables are found in three lettuce studies. Greenhouse-grown and salinized plants treated with either root or combined foliar and root application of PHs ‘Trainer’ at the rate of 2.5 mL biostimulant L^−1^ showed higher shoot fresh weight compared to the salt stressed and unstressed control.

Furthermore, treated plants showed a higher root growth in length and diameter, which, in the combined treatment, translated into a 76% higher root surface. This, coupled with the 25.8% higher photosystem II quantum efficiency (Fv/Fm) obtained across biostimulant treatments [148], shows the potential of PH in ameliorating stresses.

Similar results were recorded in a 2017 greenhouse study, but whilst the same ‘Trainer’ biostimulant was employed as a foliar spray at the rate of 2.5 mL of biostimulant L^−1^ solution, it was augmented with a microbial biostimulant that may have interacted with the product. Nevertheless, a better tolerance to the alkaline (pH 8.1) nutrient solution was recorded with the same metrics (shoot fresh weight, root surface, and PSII quantum efficiency) [21].

Lastly, both lettuce and baby lettuce grown in non-fertilized plots and treated with weekly foliar sprays of the aforementioned biostimulant at a rate of 3 mL of biostimulant L^−1^ showed a comparable yield to lettuce fertilized with 10 kg ha^−1^ of N [149,150]. The treated lettuce plants also showed a better physiological status than their untreated control, as shown by higher soil plant analysis development (SPAD) values and enjoyed better stress protection measure by the higher lipophilic (+23.3%) and hydrophilic (22.4%) antioxidant activities [149].

Table 2 shows an overview of the effects biostimulant substances have on stressed leafy vegetables.

### 4.2. Implication of Biostimulant Substance Treatments on Leafy Green Growth and Yield

SWEs seem to significantly enhance the agronomic performance of leafy vegetables. In a 1992 growth chamber spinach study [151], a foliar spray of the *A. nodosum* extract ‘Goemar GA 14’ showed significantly higher fresh matter production compared to the untreated controls by 12 to 15%. The weight increase was linked to an increase in spinach leaf area and not leaf number, which is the same result that was obtained in 2018 by Rouphael and collaborators [55], who tested ‘Amalgerol’, a blend of *A. nodosum* and oils, and the ‘Kelpak’ seaweed extract in greenhouse conditions. A weekly foliar application of both products at the rate of 3 mL of biostimulant L^−1^ of solution resulted in equally increased yields by 48.3% and leaf area by 15.4%. The results were linked to higher SPAD values, thus indicating better photosynthetic performance, and better potassium and magnesium nutrition. The results are shared—though a lower (14.4%) increase in yield was obtained—with La Bella and collaborators [152], who similarly employed the *E. maxima* extract ‘Kelpstar’ in a protected environment.

Interestingly, the same results were not obtained in a separate growth chamber study by Xu et al. [147] using a different, but still *A. nodosum*-based, biostimulant, ‘Stimplex’. No significant differences were denoted in leaf number and leaf area between the treatments at the manufacturer suggested rate of 5 mL of biostimulant L^−1^ of solution, thus furthering the argument about the different seaweed products being variably effective.

The *E. maxima* extract ‘Kelpak’ on lettuce showed similar results to what was obtained on spinach, with the SW treatments producing the highest marketable yield at the highest (equivalent to 30 kg N ha^−1^) fertilization levels, compared to a control and two biostimulants, one being PHs ‘Trainer’ and the other being a tropical plant extract, and generally significantly higher than the control results at lower fertilization levels [149].

A 2013 assessment [153] showed that foliar sprays at 2.5 mL L^−1^ of the ‘Trainer’ PH did not seem to have meaningful effects on lettuce grown in a floating system with full strength nutrient solution. The findings seem to be confirmed by a later study [154] though, and contrary to biostimulant *ethos*, the authors preferred to replace the nutrient solution inorganic nitrogen with the Amino16 PHs. Nevertheless, what emerged from this study is that crop uniformity was substantially increased by the application of the product, with lettuce in the 200–249 g weight class being significantly higher in number compared to the inorganic N supplementation (55% vs. 24–28% of control, which provided nitrogen in inorganic form).

Xou and Mou [155] provided proof of an increase in fresh biomass due to fish-derived PHs by recording significantly higher leaf numbers (+27%), and shoot and root weights; whilst leaf numbers could be a reasonable indicator of higher marketable yields, they are not an absolute measure. However, Di Mola and collaborators later recorded increases in the yields of tunnel grown baby lettuce after treatments with ‘Trainer’, which were significant at below-and-above-optimal levels of fertilization (0, 10, and 30 kg N ha^−1^). Significant differences were also denoted in the growth-related parameters such as leaf area index (LAI) and SPAD values, though no exact figures were published [149].

However, more recent evidence has shown that results may differ from the genotype, dosage, and application mode. By greenhouse-testing two varieties differing by shape and pigmentation in a floating raft system, Cristofano and collaborators [156] found that the green butterhead ‘Ballerina’ cultivar favored nutrient solution applications of the ‘Trainer’ biostimulant, with the 0.15 mL L^−1^ nutrient solution treatment showing an 82.7% higher yield compared to its control. Conversely, the red crisphead ‘Canasta’ preferred combined foliar and nutrient solution treatments, which at the rate of 3 mL L^−1^ foliar and 0.35 mL L^−1^ nutrient solution, promoted a 55.4% increase in fresh yield.

Two 2019 greenhouse-rocket studies by both Caruso and Di Mola [157,158] showed a different trend; in the former study and when averaged over two successive crop cycles (winter and winter–spring), plants treated with ‘Trainer’ PHs at the 3 mL L^−1^ rate showed a significant (11.4%) increase in marketable yields, with the higher recorded values of SPAD in line with the consulted literature [157]. In the latter, also employing the same formulate, a 33% increase in baby rocket’s marketable yield was found, even when averaged across nitrogen treatments and successive harvests [158]. The results were also confirmed by a subsequent study by Giordano and collaborators [159], who tested a 4 mL L^−1^ dosage of the same biostimulant and obtained a 50.7% increase in rocket yield when averaged across three consecutive harvests.

Two different spinach studies using the ‘Trainer’ biostimulant showed similar yet distinct results [55,160]; while the latter found significantly higher yields (57%), SPAD values, and nitrate contents compared to the untreated controls, the former found yield increases only at suboptimal levels of nitrogen fertilization (0–15 kg N ha^−1^), but, nevertheless, the spinach yield at the 15 kg N ha^−1^ level was not significantly different than the 30 kg ha^−1^ control treatment. A more recent study by Di Mola and collaborators [161] shed light over the previous results obtained by the previous authors by also trialing spinach growth and nitrogen applications and finding that foliar applications of ‘Trainer’ at the rate of 4 mL L^−1^ increased yields by 23.5%, but, interestingly, improved N-use efficiency and N-uptake efficiency compared to the untreated plants, by 18.8 and 73.3%, respectively.

Evidence found in the literature regarding the use of HSs on lettuce, point to it being generally effective in increasing growth performance, though some specifications are to be made. Dosage rates were evaluated in a 2010 study [162] on open field-grown lettuce sprayed with HAs at the rate of 2 and 4 mL HA L^−1^. Across two growing seasons, the best performing 4 mL L^−1^ group recorded a 75.2 and 30.3% yield increase, respectively, compared to the untreated control. The treatments also showed, on average, 30.3% higher leaf numbers, a result shared with what was found by Hernandez and collaborators [163]. Moreover, the results were coupled with higher dry weights and lower nitrate contents than the control, and the higher dosage also yielded higher potassium (+16 and +12.2%) and phosphorous (+24 and 12.9%) contents in both growing seasons [162].

Similar results were obtained in two other lettuce studies by Mirdad and Kiran [164,165], who both tested combinations of fertilization and HSs applications. The former author tested soil applications of Has at varying rates (30 through 90 L ha^−1^), which were evaluated at two different nitrogen fertilization levels representing non fertilized and optimally fertilized. In the non-fertilized plots, 90 L ha^−1^ HA determined an increase in growth parameters such as stem length (+28.8%), root length (+38%), shoot fresh weight (+150.8%), and dry weight (+159%) and also an increase in leaf nitrogen (24.15%), potassium (67.1%), magnesium (29.6%), and manganese (+75%) contents.

Interestingly, in both studies, a growth regression was denoted when combined HA and fertilizer were applied. Shahein and collaborators [166] also found differences in the cultivar response to biostimulant applications, as the two tested lettuce cultivars ‘Dark Green’ and ‘Big Bell’ reacted differently to mixtures of HSs derived from different matrices and supplied as a substitution for 50% of the mineral fertilizer. In fact, the foliar treatments of compost-derived humic substances elicited significantly higher fresh weights only in ‘Big Bell’, with a 31.3% increase in fresh yields across two growing seasons. Similar results to lettuce plants were obtained in 2015 [167] on spinach plants treated with foliar sprays of HA at the rate of 4.76 and 9.52 L ha^−1^. Averaged across N fertilization, HA treatments were the highest yielding across two different growing seasons, with the highest concentration being most effective at increasing the plant fresh weight (+24.6 and 63% in the 2013 and 2014 seasons). Still, and contrary to lettuce studies, no macronutrient differences were denoted between treatments for phosphorous and potassium, except for nitrogen (+9.7 and +9.6% for each season).

Table 2 shows an overview of the growth and yield-promoting effects biostimulant substances have recorded on leafy vegetable crops.

### 4.3. Leaf Quality Modulation after Biostimulant Applications

Rouphael and collaborators [55] found, in a 2018 greenhouse study, that weekly foliar treatments of the ‘Kelpak’ extract and the combined seaweed and oil extract ‘Amalgerol’ at the rate of 3 mL L^−1^ increased spinach leaves’ potassium and magnesium contents by 25.6 and 20.1%, respectively. The same study also found a 30.7% increase in leaf phenolics and a 79.1% increase in total ascorbic acids. However, both biostimulants also recorded an average 41.1% increase in leaf nitrate contents, which, while still remaining under the EU limit of 3500 mg kg^−1^, was just 4.3% below.

Later research on greenhouse-grown lettuce plants found significantly increased leaf succulence by 7.8% and carotenoids content by 16.8% after plants were foliarly treated with a 3 mL L^−1^ solution of the ‘Kelpak’ extract. The total ascorbic acid content increase was found to be N-fertilization-dependent, as it was significantly (+33.6%) higher than the untreated control only in non-fertilized plots [149].

The PH ‘Trainer’ was the treatment of choice for the consulted literature on this particular biostimulant grouping.

When averaged across four fertilization levels (0–10–20 and 30 kg ha^−1^), tunnel-grown baby leaf lettuce treated with a 3 mL biostimulant L^−1^ foliar spray showed 16.4% higher leaf succulence compared to the untreated controls and increased leaf pigment contents, with carotenoids and chlorophyll being 11.6 and 12.8% higher, respectively [149].

The same authors also noted that the antioxidant activity increases were nitrogen-fertilization-dependent as lipophilic and hydrophilic activity were 23.3 and 22.4%, respectively, higher in the plants grown in unfertilized plots, whereas the hydrophilic activity was 40.6% higher at the highest fertilization level.

Furthermore, Cristofano and collaborators [156], who tested the same formulation on lettuce grown in a floating raft system in greenhouse conditions, found the increases in lettuce quality parameters to be genotype-, treatment dosage-, and modality-dependent. In fact, of the two tested cultivars, the red butterhead ‘Canasta’ recorded the highest hydrophilic antioxidant activity (+21.9%) and total ascorbic acid contents (a 5.6-fold increase) when both the foliar and nutrient solution treatments were applied, whilst the green butterhead ‘Ballerina’ recorded its highest contents of ascorbic acid (+51.2%) when only foliarly treated.

When weekly foliar sprays of the same formulate at the 3 mL L^−1^ rate on greenhouse-grown spinach were investigated, a 36.4% increase in potassium contents was found, coupled with a 30.7% increase in leaf phenolic contents and a 79.1% increase in the total ascorbic acid content [55]. A later experiment, also on spinach, was undertaken by Carillo and collaborators [160], who also investigated nitrogen fertilization (0–15–30–45 kg ha^−1^) and ‘Trainer’ applications at the 4 mL L^−1^ rate. Researchers found that across fertilizer levels, the treated plants showed 12.9% higher leaf phosphorous, 12.8% higher calcium, 10.3% higher magnesium, but 10.8% lower polyphenolic contents. Moreover, a 43.5% increase in amino acid content was denoted, of which the essential amino acids glutamic acid and alanine received a boost of 17.2 and 39.7%, respectively.

Consistently with previous studies and when averaged across consecutive winter and winter–spring crop cycles, foliar sprays of ‘Trainer’ on rocket yielded plants with higher phosphorous, calcium, and polyphenolic contents (+11.9, 9.5, and 10.8%, respectively), but also of higher ascorbic acid contents (+11.9%) and hydrophilic (+18%) and lipophilic activities (34.4%) [157]. However, the same results were not obtained by a subsequent study [159], using the same biostimulant (albeit at the higher 4 mL L^−1^ dosage regime) and greenhouse conditions, also in the winter–spring cycle. After three consecutive harvests, researchers found that the leaf potassium increase was only significant at the third harvest, with a 44.8% increase and, likewise, magnesium, which increased by 43.4%. The leaf tissue calcium increase was more consistent, with a 30.6% increase when averaged across successive harvests.

Lettuce plants treated with HSs showed no significant increases in total soluble solids content in two separate studies [162,166]. However, Fawzy found that across two growing seasons, two bi-weekly foliar applications of HAs at the rate of 4 mL L^−1^ on open field-grown lettuce increased leaf phosphorous, potassium, zinc, and magnesium contents by 18.5, 14.2, 9.9, and 30%, respectively, and consistently decreased the leaf nitrate content by 19.6% [162]. Aslam and collaborators [168] also tested the foliar application of 10% HA on spinach. The results from the open-field trial suggested that treatment repetition may be an important factor when product quality is a consideration: the recorded increases in phenolics (28.9% on average) and carotenoids (76.5%) were only deemed significant after the second and third treatments, respectively.

Table 2 shows an overview of the leaf quality modulation after biostimulant applications.

## 5. Nightshades (*Solanaceae* Juss.)

### 5.1. Biostimulants Substances to Increase Nightshade Resilience to Stress

Most research is focused on pepper (*Capsicum annum* L.) and tomato (*Solanum lycopersicum* L.) plants, and it is shown that excess salinity causes decreases in many physiological and growth parameters from germination to fruit yield. From the consulted literature, nutrient solution application of soluble silicon is the most studied and varied by rate (0.5 through 3 mM) and type i.e., engineered nanosilica vs. silicate salts such as sodium and potassium silicate.

Salt stress studies show that low (0.5 mM) silicon supplementation can roll back germination percentages up to control levels, when up to a 150 mM NaCl stress is applied [169]. The increased plant growth parameters of salt stressed tomato plants such as plant fresh and dry weights [44,170] are further outlined by the increased mineral contents [171]. The ameliorative effect of silicon supplementation has been found to be cultivar-dependent, as a growth chamber study by Wasti and collaborators [172] on two tomato cultivars, ‘Rio Grande’ and ‘Moneymaker’, illustrated that calcium silicate treatments were more effective, and at lower dosages (2 mM vs. 4 mM), in the former cultivar at reducing the effects of 100 mM NaCl stress. Silicon supplementation at the rate of 1 mM significantly increased tomato yield compared to the non-stressed and salt-stressed controls (12.4% when averaged across salt treatments) and significantly decreased the blossom-end rot symptoms by 46.5% [171].

Studies on pepper plants show similar results: in a 2018 growth chamber study [45], it was found that salt stressed plants supplemented with 2 mM soluble silicate showed a higher dry weight (results also found by Manivannan and collaborators [173]), leaf area, and photosynthetic rate compared to the stressed control. Again, the efficacy of the treatment was found to be cultivar-dependent, as it was more pronounced in the salt-tolerant ‘Karaisoli’ versus the sensitive ‘Demre’.

Silicon treatments were also proven to be beneficial in the case of drought stress. The treatments improved the growth of drought-stressed (simulated through polyethylene glycol 6000) tomato plants in a genotype dependent manner, by differently modulating the stress response mechanisms. In particular, researchers found that silicon supplementation was more advantageous to the drought sensitive ‘FERUM’ line, compared to the resistant ‘LA0147’ line [174]. The effects of silicon supplementation also include increased tomato plant shoot and root growth, chlorophyll contents, and quantum efficiency compared to drought-stressed controls; increased transpiration rates versus the control; and improved leaf relative water content (RWC) in stressed pepper plants, whilst maintaining nitrogen metabolism, which manifests as higher nitrate reductase activity [46,175,176,177].

*A. nodosum* commercial extracts, ‘Rygex’ and ‘Super Fifty’, were tested in a greenhouse experiment on nutrient deprived (70% of the basic nutrient solution) and salinized ‘Microtom’ tomato plants in a 2018 study [178]. Both treatments mitigated the effects of salinity by increasing the potassium, calcium, and nitrate contents and lowering the sodium and chloride contents compared to the stressed controls; however, when averaged across nutrient and salt stresses, the ‘Rygex’ treatments caused a reduction in the fruit fresh weight of 17.1%. GC-MS analysis conducted in the previous research showed that the two biostimulants, whilst produced from the same biomass, yielded different products. The discovered differences in the amounts of bioactive compounds and minerals, with ‘Super Fifty’ delivering four times more potassium and magnesium, whilst ‘Rygex’ delivered seven times more calcium, are indicative of some variation between the two treatments.

In a later study, the same two commercial products were found to be successful in open field conditions at priming tomato plants before a saline stress was applied: biostimulant drip-irrigated plants were found to have improved water efficiency, improved shoot-to-to root ratio, and were ultimately better yielding (48.7 and 70% for Rygex and Super Fifty, respectively) than the untreated controls [74]. An *A. nodosum* extract was also tested in salt-stressed pepper plants in a greenhouse study [179]. Compared to the salt stressed (100 mM NaCl) controls, the plants treated by drip irrigation with the biostimulant showed a significantly higher dry matter and fruit yield (though the exact figures were not published) and, interestingly, a reduction in the plant proline content and ROS-scavenging mechanisms such as SOD and CAT, which may suggest a better plant oxidative state.

Drought-stressed tomato plants also showed similar results, with foliar treatments of the *Ascophyllum nodosum* extract ‘Bio-algeen S92’ eliciting the production of antioxidants and phenolics and with treated plants showing a 21.6% higher leaf area and a 20.3% leaf chlorophyll content. Moreover, the plants treated with the product had a significant boost in fruit yield of 65.4% compared to the stressed controls, and also manifested the highest fruit lycopene and flavonoid contents [180].

Tomato plants grown in a growth chamber with an iron-derived nutrient solution (4 μmol, or one tenth of the full-strength solution) and subject to two weekly treatments of PH ‘Trainer’ at the rate of 3 mL L^−1^ showed a 50% increase in biomass when compared to the untreated plants [127]. Iron reductase activity was reverted to the condition of control plants supplied with a complete nutrient solution when the biostimulant was applied, whereas the untreated, iron deficient controls registered a 70% increase. Interestingly, the iron stored in biostimulant-treated shoot tissues in the low iron group was almost two-fold higher than the untreated control.

Francesca and collaborators [88] employed the PH ‘CycloFlow’ on open field-grown tomato plants, to which a 50% water deficit was applied. Root drench treatments yielded plants with 51% more pollen viability and 70% higher fruits per plant, which were, on average, weighing 95% more. All in all, the researchers obtained a six-fold increase in the final yield compared to the untreated controls.

A 2004 study [181] on salt-stressed tomato plants showed that HA supplementation to the growing medium at a rate of 500 through 2000 mg HA kg^−1^ significantly improved seed germination, shoot length, and leaf numbers. Moreover, HAs increased both the shoot and root micro (copper, iron, manganese, and zinc) and macronutrient (nitrogen, phosphorus, potassium, sulfur) contents in a dose dependent manner, with the 1000 mg kg^−1^ dose being the most effective.

A later greenhouse study [182] confirmed the findings, but also demonstrated that repeated root applications of HAs at the rate of 750 mg L^−1^ significantly increases the number of fruits per plant and fruit mass, thus increasing, on average, the fruit yield by 27.5%. Conversely, increasing HA treatments significantly lowered quality traits such as TSS and fruit juice EC (an average 11.1 and 12.2% reduction, respectively) compared to the saline-stressed controls.

The results obtained on tomato plants were also found on hot pepper (*Capsicum annum* L.) plants: the application of HAs yielded better growth and yield parameters compared to the stressed and non-stressed controls, while also improving on the plants’ nutrient status (higher nitrogen, phosphorous, and potassium) [183,184]. The best results were obtained by adding calcium nitrate to the HA treatments. However, it has to be noted that two consecutive studies (2016 and 2017) from Bacilio and collaborators [184,185] under greenhouse and field conditions on pepper cultivars ‘Jupiter’ and ‘Ancho San Luis’ provide evidence of HA treatments being particularly beneficial only to salt-susceptible cultivars at high dosages.

Table 3 shows an overview of the effects biostimulant substances have on stressed solanaceous crops.

### 5.2. Implication of Biostimulant Substance Treatments on Nightshade Green Growth and Fruit Yield

SWEs determine a variety of growth-promoting effects on solanaceous plants. Tomato and pepper seeds treated with such products showed a higher germination rate, lower germination time, and amplified germination energy [186,187,188]; Muthu-Pandian Chanthini and collaborators [186] also found, in 2019, that the tomato seeds treated with pure *Chaetomorfa antennina* water extract gave rise to pot-grown plants that exhibited higher growth parameters such as 16% higher plant height, 110.5% more branches, 40.1% higher leaf numbers, and were, ultimately, 135.9% higher yielding than the untreated counterparts. Renaut and collaborators [189] found bi-weekly ANE ‘Stella Maris’ treatments to increase the fruit number in tomato plants (this one amended with hen manure) and pepper plants by 46 and 195% respectively; the tomato plants did not result in an increased average fresh weight, whilst the pepper plants recorded a 35% increase, and also increased root and shoot fresh weights.

The mode of application and cultivar selection seem to be important when deciding to employ seaweed extracts. When Ali and collaborators [190] grew greenhouse tomato plants with a 0.5% foliar spray of an ANE, they found that it was more effective at increasing yields than the substrate drench treatment that brought more fruit bearing clusters (+81% compared to control), higher (+54%) per plant yield, and heavier fruits (55% in the >70 g category vs. 18% of the control). A later study [191], also in greenhouse conditions, confirmed the efficacy of ANE ‘Stimplex’ foliar treatments at the 0.5% rate, which averaged a +71.5% and +80.9% yield increase in tomatoes and peppers, respectively. Dobromilska and Gubarewicz [192] grew tomato plants in greenhouse and open field-conditions using ‘Bio-algeen S90’ over three growing seasons at the rate of 0.3% at four different growing stages. When the plants were sprayed three times, at the two-three true leaves stage, before planting and the beginning of flowering, a 49.5% yield increase was recorded, coupled with increased fruit nitrogen, phosphorous, and potassium contents and increased photosynthetic parameters.

Similar results were obtained by Li and Mattson [193], who found that 20 mL L^−1^ foliar treatments of ANE ‘Stimplex’ elicited a 15.9% increase in tomato transplant weight compared to a −43% decrease in the drench group, both compared to the untreated control.

Nevertheless, it is plausible that some dosage issues may have been at play, especially in the latter study, as either *A. nodosum* treatments via fertigation on open field and greenhouse-grown tomato plants and combined pre-transplant soak and foliar spray on both tomato and pepper plants recorded significantly higher fruit yields [54,86].

Genotypical variation may also be a factor at play. Khan and collaborators [194] found that greenhouse grown pepper cultivars sprayed with ANE ‘Wokozim’ at 15-day intervals at the rate of 2 and 4 mL L^−1^ behaved differently, as the ‘Sven Rz’ cultivar showed an 83% increase in yields, whereas ‘Red knight’ showed a 46.4% increase, both compared to the respective untreated controls. More recent research conducted by Melo and collaborators [195] recorded in-between results by ‘Elisa’ peppers sprayed with a 0.5% solution of ‘Reabilit Algas’. The mixture of *Kappaphycus alvarezii* and *Sargasum vulgare* increased the 1000 plant yield by an estimated 68.8%, compared to its untreated control.

Conversely, Arthur and collaborators [196], while also testing three pepper cultivars (yellow ‘Orobelle’, red ‘Indra’, and ‘King Arthur’) using ‘Kelpak’, found increases in fruit numbers and average fruit weight to be significant only in ‘Indra’ and with combined pre-transplant soak and foliar spray.

Genotype-dependent efficacy is not only limited to pepper plants, as out of six eggplants (*Solanum melongena* L.) cultivars grown in open field conditions and sprayed with ANE Göemar BM-86 at the rate of 1.5 L of biostimulant ha^−1^ only ‘Epic’. ‘Flavine’ and ‘WA 6020 F1’ registered significant increases in yield across two growing seasons. ‘Epic’ and ‘Flavine’ had a significantly higher fruit number, whereas ‘WA 6020 F1’ registered higher fruit weight [197].

Treating tomato plants with PH products increased tomato growth and yield in four separate instances [86,198,199,200]. Foliar treatments at a rate of 3 and 5 mL L^−1^ with the commercial PH ‘Trainer’ significantly increased growth in tomato plants grown in either open field and greenhouse studies. In open-field, Caruso and collaborators recorded a 14.6% total aerial biomass, and 18.6% higher yields stemming from 19.3% higher fruit numbers in ‘Vesuvian Piennolo Tomato’. Similar results were obtained by Colla and collaborators in greenhouse conditions [198,199]. Open field-grown plants treated with the animal-biomass-derived ‘Pepton’ recorded dose-dependent increased growth parameters such as height, stem diameter, and 31.1% higher leaf number at the highest supplied dosage of 300 g ‘Pepton’ L^−1^. In a similar dose-dependent way, ‘Pepton’ treatments also significantly increased tomato yield, which, at its highest dosage, reached an estimated 27.5% increase compared to the untreated controls [86].

Genotype and dosage-dependent efficacy was also proven by Rouphael and collaborators [200] in greenhouse-grown tomato plants, by testing two treatment rates of 2.5 and 5 mL L^−1^ on tomato cultivars ‘Akyra’ and ‘Sir Elyan’; the researchers found the highest concentration to be the most effective in improving both plant growth and average tomato yield (+21.3%, compared to the control), but also to variably increase yield parameters. In fact, at the best performing treatment rate, ‘Akyra’ recorded a 13.9% higher fruit number, whereas ‘Sir Elyan’ bore fruits that were 28.7% heavier than the control treatments.

Dose-dependent results were also obtained in a growth tunnel study on alfalfa-based treatments on hot pepper plants; plants sprayed with two dosages (2.5 and 5%) of a solution of alfalfa hydrolysate showed an increased fruit number, which was highest at the elevated dosage [201].

There is a substantial body of evidence confirming the validity of HS treatments on nightshade plants. The effects include an increased rate of seed emergence in tomato and eggplant plants and seedling growth in tomato and pepper plants when HA was added to the growing medium at low concentrations (0.5 g per L^−1^ and 0.2%, respectively [95,202]); effects also include increased plant vegetative growth parameters such as the fresh and dry biomass of shoots and fruits, LAI, and plant height [203,204,205,206]. HA application was also found to increase the leaf nutrient concentration of nitrogen, phosphorous, and potassium of tomato plants [207] and nutrient transfer from the growing medium in tomato and eggplant plants [95].

The most glaring effect of the treatments on nightshades is the increased fruit yield [203,204,205,206,207,208,209,210], which is usually dose and application mode-dependent.

From the consulted tomato studies in either open field or greenhouse conditions it is found that optimal soil applications soil application may lie at around 100 to 200 L of HS per hectare. Abou Chehade and collaborators [208] who tried the former dosage regimen (100 L ha^−1^), recorded increasing fruit yields in a greenhouse by 18.1%, whereas Asri et al. [207] found the ranges between 160 to 200 L ha^−1^ giving rise to increased yields (+35.2%), leaf macro, and micronutrients levels (nitrogen, phosphorous, potassium, iron, zinc, and manganese). The evidence also seems to suggest that when foliar spray and substrate drench treatments are pitted against each other, it is usually the former being the most effective. Tomato and pepper plants grown in greenhouse and open field conditions, respectively, and sprayed with 20 mL HA L^−1^ recorded higher yields (+27.5 and 29.5%), due to an increased mean fruit weight (+30.4 and 22.5%) and, in the case of tomato plants, fruit number (+30.4%) [209,210].

Yield and growth increases, consistently with other studies, showed a tendency to decrease at higher dosage levels [205,209] and there is also still evidence of the treatments not being effective in increasing yields in some cases.

A three-year investigation by Suman and collaborators [209] found that adding 0.5 L humic acid ha^−1^ via fertigation to open-field-grown tomato plants did not enhance growth and yield when fertilization was 100% of the recommended dosage, which is also consistent with what Monda and collaborators [211] recently found. When fertilization was 80% of the recommended dosage, it performed significantly better than its untreated control (12.6% higher yielding), and statistically equal to the 100% fertilization group; the same results were also recorded when 25 and 50 mg of HAs were added to a full-strength nutrient solution [212], which may point to differences in either the plant, the experimental setup, and/or the HA source material and dosage.

When testing commercial HA product ‘Solum H80’, Ibrahim and collaborators [204] found that there is a significant degree of variance from cultivar to cultivar regarding the effectiveness of the treatments. Open-field-tested pepper cultivars ‘Barbero’, ‘Ferrari’, and ‘Imperio’ treated with commercial HS ‘Solum H80’ at the rate of 1.5 g L^−1^ recorded 15.7, 7.2, and 14.1% yield increases, respectively, when compared to the untreated plants, but more interestingly, also recorded differences in the yield parameters. ‘Barbero’ and ‘Imperio’ had more and bigger average fruits, whereas ‘Ferrari’ produced the same amount of fruit that was higher in weight compared to the untreated counterpart.

Lastly, and worth noting, research carried out by Hartz and Bottoms [213] in the 2008 and 2009 growing season on tomato plants grown in open field conditions found no significant differences in either growth and nutrient application between five commercial HAs formulations at the rate of 1.1 and 3.4 kg HA ha^−1^ and an untreated control. The authors attributed the cause of this behavior to the doses being insufficient for the biostimulant effect.

Table 3 shows an overview of the growth and yield-promoting effects biostimulant substances have recorded on solanaceous crops.

### 5.3. Nightshade Fruit Quality Modulation after Biostimulant Applications

Treating greenhouse-grown tomato plants with ‘Siliforce’, a commercial formulation of orthosilicic acid at the rate of 300 mL of formulate ha^−1^ brought an increase in fruit firmness, but only when the product was applied one day before harvest [24]. Such increases may nevertheless come with disadvantages: in an open field study, the treated fruits showed significantly less total soluble solids content and total acidity [214]. This is coupled with the sometimes excessive dosage regimens. In the consulted literature, an instance was found where the authors suggested silicon amendments of 400 kg ha^−1^ of silicon salts (calcium, potassium and sodium silicate) [215].

Such a proposition may render silicon treatments unpalatable to those who want to increase tomato fruit quality.

Tomato and pepper plants treated with SWEs yielded fruits that were higher in vitamin C and total soluble solids [180,186,216,217]. Murtic and collaborators [180] found foliar treatments of *A. nodosum*-based ‘Bio-algeen S92’ on greenhouse-grown tomato plants at the 0.2% concentration to increase the fruit total soluble solids, phenolic, flavonoid contents, and ferric reducing antioxidant activity (FRAP) by 3.1, 10.8, 10.5, and 10.2%, respectively, compared to the untreated controls [180].

Similar results were also obtained in separate studies using different source materials, such as undiluted *Chaetomorpha antennina* water extract, 5% *Kappaphycus alvarezii* extract, and *Sargassum johnstonii* [216,217]. Interestingly, it was noted that the root-zone drench treatment of *Sargassum johnstonii* extracts was more effective at increasing TSS, fruit phenolic contents, and lycopene and at lower concentrations, compared to foliar treatments [216].

Nevertheless, not all the literature seems agree on SWEs providing beneficial effects to fruit quality. No quality parameter improvements were recorded when Colla and collaborators [199] applied *Ecklonia maxima* extract ‘Kelpak’ at the rate of 3 mL L^−1^ on greenhouse-grown tomato plants; similar results were obtained by Di Stasio, who similarly tested ANEs ‘Rygex’ and ‘Super Fifty’ and only found increases in fruit calcium, potassium, and magnesium contents (31 and 22%, 17 and 45%, and 32%, respectively) and essential amino acids content [178]. Still, SWEs may found utilities at the post-harvest level, as increased fruit firmness retention during cold storage, coupled with a lesser oxidative increase in fruit TSS were recorded in pepper fruits [194,218]

Three instances of quality improvements using PH biostimulants on tomato plants were found in the available literature, and in each instance, the ‘Trainer’ legume-derived PH was employed [198,199,200].

Both greenhouse and field studies found that foliar treatments every 7–10 days of such product in the range of 3 and 5 mL of formulate L^−1^ consistently increased the fruit quality parameters with an average 11.7% increase in the total soluble solids across the three studies being the most repeatable effect across the literature. Tomato fruit antioxidant activity increases may be a factor of application rates, as Rouphael et al. [200], who employed 5 mL L^−1^, recorded increases in lipophilic activity of 260% and hydrophilic activity of 61.9% across the two tested cultivars ‘Akyra’ and ‘Sir Elyan’, vs. the 24.6% hydrophilic activity recorded by Colla and collaborators [199], who administered a rate of 3 mL L^−1^ on the same crop.

Other fruit quality parameters enhanced by treatments include increased potassium, ascorbic acid, and lycopene contents, the latter of which was found increased in a field study by Caruso and collaborators [198] by 106.2% over its control.

Foliar alfalfa–hydrolysate treatments at the rate of 25 mL L^−1^ on hot pepper plants grown in a growth tunnel were the most effective at increasing pepper phenol concentration (+44.8 and +140.2%), FRAP antioxidant activity (+36.8 and 27.1%), and ascorbic acid (16.1 and 153.4%) contents in red and green fruits, respectively; nonetheless, the highest tested dosage of 50 mL L^−1^ substantially increased the capsaicin concentration of red peppers by 598% [201].

From the consulted tomato plant studies, there is no consensus for HSs to elicit significant increases in quality parameters. Both Abou Chehade, Asri, and their collaborators [207,208] found that delivering HS to the soil in either greenhouse or open field conditions, respectively, at a rate of 40 through 200 L hectare^−1^ did not substantially increase any tested fruit quality indicator (titratable acidity, total soluble solids, ascorbic acid, lycopene, phenolic contents an antioxidant activity), save for a single-year when a 10.3% increase in titratable acidity was recorded by the latter authors.

Conversely, foliar treatments of HA on greenhouse-grown tomato plants at a rate of 20 mL L^−1^ increased ascorbic acid by 50.3% and total soluble solids by 18% when averaged across the experiment’s two growing seasons, thus indicating that treatment modality may be a factor when HSs are used for product quality improvement [210]. The available research on pepper plants paints a different picture, with open field studies and greenhouse studies both indicating that foliar treatments of either FA at 6% [219] and HA ‘Solum H80’ at the rate of 1.5 g L^−1^ [204] were the most effective at increasing the ascorbic acid contents of pepper fruits.

Ibrahim and collaborators [204] also found out that the increase in quality parameters were cultivar-dependent, as the three tested cultivars ‘Barbero’, ‘Ferrari’, and ‘Imperio’ recorded a respective +11, +6, and +8% increase in vitamin C contents, a +14, +8, and +10% increase in titratable acidity, and a +18, +7, and +10% increase in the fruit total soluble content. Dosage-dependent product quality improvements were also denoted in open field-grown hot pepper plants. Out of the four tested soil application regimens (50–200–350–500 kg ha^−1^), the 350 kg ha^−1^ registered an increase in antioxidant activity of 22.3% and an increase in fruit capsaicin contents by 36.8%, whereas lycopene content was highest at 200 kg ha^−1^ by +43.3% and beta carotene at 350 kg ha^−1^, with an 89.1% increase [220].

Table 3 shows an overview of the nightshade fruit quality modulation after biostimulant applications.

## 6. Conclusions

The ever-more pressing issue of climate change and the effects that agriculture has on the environment has posed the dilemma of rapidly finding new answers for the sustainable intensification of crop practices.

Whilst these problems are multifaceted and may require a complete rethinking of how agriculture should be managed worldwide, the introduction of biostimulant substances have brought a valid interim solution toward the future of agriculture. These substances derive from, or are generated by, industrial waste or waste biomass, therefore, limiting the recourse to newly and wastefully generated fertilizers. Furthermore, they prove their worth by increasing plant growth, reducing plant stress, and increasing produce quality at low dosage applications, thus earning their namesake.

However, there is still space for arguing about some of the sore points that have been found in the consulted biostimulant literature. The incredibly wide selection of source materials, from seaweed species through the plethora of waste streams that can be made into humic substances, creates a variety of products that contain a plethora of active ingredients. The same active ingredients have been hard to discover, certainly not helped by the great number of often proprietary production methods, and the list of the ones we currently know is by no means exhaustive and still leaves some doubts.

Agronomic factors such as cultivar selection and biostimulant management i.e., how much biostimulant to use, when to use it, where to use it (greenhouse or open field), and in which modality it is administered (foliar, drench, seed treatment, nutrient solution), sometimes are the make-or-break decisions that may or may not express the crops’ and products’ full potential, and have to be carefully considered.

The picture depicted here shows the need for interdisciplinary biostimulant research: products need to be scrutinized at the molecular level, which could be performed by the way of fractionation or separation; rapidly and repeatedly screened via metabolomic, genomic, and physiological analysis; and then tested against widely used crop benchmarks in order to assay their performance. Thus, a top-down approach might be needed going forwards, and judging from the consulted literature, it is currently happening, and it is a welcomed change for agriculture worldwide.

## Figures and Tables

**Table 1:** An overview of the abiotic stress amelioration, growth improvement, and fruit quality enhancement by biostimulant substances on cucurbits.

Abiotic Stress Amelioration							
**Cucurbit**	**Growing Conditions**	**Biostimulant Substance**	**Application Method**	**Dosage**	Intervention Time	Effect of Biostimulant Substance	References
**Cucumber**	Laboratory and Greenhouse	Silicon as engineered nanosilica	Via irrigation	100, 200, and 300 mg L^−1^	50% before planting and 50% 7 days after planting	100 and 200 mg L^−1^ treatments were most effective at increasing germination parameters and seedling growth under Saline stress.	Alsaeedi et al., 2018
	Growth chamber	Silicon as silicic acid	Nutrient solution	1.4 mM of Silicon		Silicon ameliorated iron and partially ameliorated Zinc and Manganese deficiency symptoms.	Bityutskii et al., 2014
	Growth chamber	Silicon as silicic acid	Nutrient solution	1.5 mM of Silicon		Silicon ameliorated salinity symptoms by increasing photosynthesis and decreasing leaf fluorescence.	Harizanova and Koleva-Valkova, 2019
	Growth Chamber	Protein Hydrolysate ‘Trainer’	Foliar	3 mL L^−1^	Two spray treatments at weekly intervals.	Treated plants showed higher iron contents and chlorophyll content.	Celletti et al., 2020
**Zucchini squash**	Greenhouse	Silicon as potassium silicate	Nutrient solution	0.1 and 1 mM Silicon		1 mM Silicon increased fruit number per plant and physiological parameters of salt stressed zucchini.	Savvas et al., 2009
	Greenhouse	Seaweed extract ‘Kelpak’ (Ecklonia maxima)	Foliar	3 mL L^−1^	Spray treatments at biweekly intervals, starting from 10 days after transplanting	Treated plants showed higher marketable yields of 12%, when averaged across salinity levels.	Rouphael et al., 2017
**Watermelon**	Greenhouse	Silicon as silicic acid	Irrigation	4 mM Silicon		Silicon treatments increased plant growth and fruit yield, and decreased salt-related oxidative stress	Bijalwan et al., 2021
**Plant Growth and Fruit Yield Enhancement**							
**Cucurbit**	**Growing Conditions**	**Biostimulant Substance**	**Application Method**	**Dosage**	**Intervention Time**	**Effect of Biostimulant Substance**	**References**
**Cucumber**	Greenhouse	Silicon as wollastonite or K_2_SiO_3_	Irrigation and soil incorporation	125 mg SiO_2_ per plant. 2–4–8 g wollastonite L^−1^ soil.	Irrigation treatments 6 days a week, from planting.	No increase in growth and fruit yield was recorded.	Dorais and Thériault, 2018
	Greenhouse	Seaweed extract ‘TAM’ (*Ulva lactuca*, *Jania rubens Pterocladia capillacea*)	Foliar	2.5, 3.5, and 5 mL L^−1^	Bi-weekly treatments during the growing season.	When used in substitution of 25, 50, and 75% of NPK fertilizer TAM elicited a 51.9% average increase in cucumber fruit yield.	Hassan et al., 2021
	Greenhouse	Humic Acid	Foliar and soil application	Foliar at 10–20–30–40 mL L^−1^, soil applications at the same rate.	Foliar and soil applications at 15 day intervals four weeks after planting.	20 mL L^−1^ foliar and 30 mL L^−1^ soil application elicited 14.9 and 14.5% yield increases.	Unlu et al., 2011
	Greenhouse	Humic Acid	Soil incorporation	0.5, 1, 3, 5 g kg^−1^ calcium salts of ‘Actosol’, and ‘Actosol’.	Incorporation before planting.	Calcium plus ‘Actosol’ increased yields by 28.7%, versus 14.4 of ‘Actosol’ alone.	Ekinci et al., 2015
**Fruit Quality Modulation**							
**Cucurbit**	**Growing Conditions**	**Biostimulant Substance**	**Application Method**	**Dosage**	**Intervention Time**	**Effect of Biostimulant Substance**	**References**
**Cucumber**	Greenhouse	Silicon as wollastonite or K_2_SiO_3_	Irrigation and soil incorporation	125 mg SiO_2_ per plant. 2–4–8 g wollastonite L^−1^ soil.	Irrigation treatments 6 days a week, from planting.	No increase in Total soluble solids, Ascorbic Acid. No difference in peel and pulp color.	Dorais and Thériault, 2018
	Greenhouse	Humic Acid	Foliar and soil application	Foliar at 10–20–30–40 mL L^−1^, soil applications at the same rate.	Foliar and soil applications at 15 day intervals four weeks after planting.	10 mL L^−1^ treatments increased total soluble sugars. 20 mL L^−1^ treatments increased antioxidant activity, carotenoid, lycopene, and beta carotene contents.	Unlu et al., 2011, Karakurt et al., 2015
**Watermelon**	Greenhouse	Silicon as silicon Hydroxide (SiOH)_4_	Irrigation	260 mL of formulate ha^−1^	Bi-weekly treatments, starting 23 days after planting.	No increase in Total soluble solids, Ascorbic Acid. No difference in peel and pulp color.	Toresano-Sánchez et al., 2010

**Table 2:** An overview of the abiotic stress amelioration, growth improvement, and fruit quality enhancement by biostimulant substances on leafy vegetable crops.

Abiotic Stress Amelioration							
Leafy Vegetable	Growing Conditions	Biostimulant Substance	Application Method	Dosage	Intervention Time	Effect of Biostimulant Substance	References
**Baby lettuce and Lettuce**	Plastic Tunnel	Protein Hydrolysate ‘Trainer’	Foliar spray	3 mL L^−1^	Spray treatments at 7 day intervals, starting from three weeks after sowing.	Unfertilized plants showed comparable yield to lettuce amended with 10 Kg ha^−1^ of N	Di Mola et al., 2019 Di Mola et al, 2020
**Lettuce**	Laboratory	Silicon as sodium silicate	In solution	6 mM Na_2_SiO_3_		Improved seed germination parameters compared to salt stressed controls.	Neto et al., 2018
	Greenhouse	Silicon as sodium silicate	Nutrient solution	1, 2, and 4 mM silicon		2 mM silicon increased shoot and root fresh weights by 71.5 and 75.2% compared to the 60 mM salt stress control.	Milne et al., 2012
	Growth Chamber	Silicon as potassium silicate	Nutrient Solution	1 mM silicon		Increased plant dry and fresh weights compared to Arsenate and Arsenite stressed controls.	Greger et al., 2015
	Greenhouse	Seaweed extract ‘Super Fifty’ and ‘Ecolicitor’ (*Ascophyllum nodosum*)	Nutrient Solution	0.4, 1, 2.5, and 10 mL L^−1^		‘Super Fifty’ increased fresh weights of salt-stressed plants up to the non-stressed control.	Guinan et al., 2013
	Greenhouse	Seaweed extract (*Ascophyllum nodosum*)	Foliar	1%	Treatments were administered at 2-week intervals	Treatments ameliorated K-deficiency stress by improving growth to control levels.	Chrysargyris et al., 2018
	Greenhouse	Protein Hydrolysate ‘Trainer’	Foliar spray and combined spray and french	2.5 mL L^−1^	Treatments were administered 5 days after transplanting, and weekly.	Saline-stressed plants recorded fresh weights comparable to unstressed controls. Combined treatments yield 76% higher root surface.	Lucini et al., 2015
**Spinach**	Laboratory	Seaweed extract ‘Improver’ (*Ascophyllum nodosum*)	Seed Priming	0.15, 0.3, 0.6, and 1.2%		Improved germination by 25% compared to heat-stressed controls. 0.6% treatment was the best performing	Neto et al., 2020
	Growth chamber	Seaweed extract ‘Stimplex’ (*Ascophyllum nodosum*)	Foliar spray, substrate drench, combined	0.5% solution for both treatments	Treatments were administered every four days	Both treatment modalities ameliorated drought stress by increasing fresh and dry weights and photosynthetic parameters	Xu e Leskovar, 2015
**Plant Growth and Yield Enhancement**							
**Leafy Vegetable**	**Growing Conditions**	**Biostimulant Substance**	**Application Method**	**Dosage**	**Intervention Time**	**Effect of Biostimulant Substance**	**References**
**Baby Lettuce**	Plastic tunnel	Protein Hydrolysate ‘Trainer’	Foliar	3 mL L^−1^	Spray treatments at weekly intervals. from three weeks after sowing.	Treatments increased yields at below and above optimal nitrogen fertilization levels.	Di Mola et al., 2019
**Lettuce**	Greenhouse	Protein Hydrolysate ‘Trainer’	Nutrient solution, foliar, and combined	0.15 and 0.3 mL ‘Trainer’ L^−1^ nutrient solution applications, foliar at 3 mL L^−1^	Foliar treatments after transplanting, and every 6 days. Nutrient solution application when refilled.	Nutrient solution only applications increased green butterhead yield by 82.7%. Combined applications increased red crisphead yield by 55.4%.	Cristofano et al., 2021
	Open Field	Humic acid	Foliar	2 and 4 mL L^−1^	Spray treatments at 4 and 6 weeks after transplanting.	The 4 mL L^−1^ treatments increased yield by 75.2 and 30.3% in the two growing seasons.	Fouad Fawzy, 2010
	Open Field	Humic Substance	Foliar, soil, and combined applications	3.8 and 3.3 L ha^−1^ soil applications and 1.9 and 1.7 L ha^−1^ foliar for compost and biogas manure-extracted humic substance, respectively.	Treatments were administered at 3, 6, and 9 weeks after transplanting.	Foliar applications of compost-derived humic substance elicited 31.3% in fresh yields in only one cultivar.	Shahein et al., 2014
**Spinach**	Greenhouse	Seaweed extract (*Ascophyllum nodosum*-based ‘Amalgerol’ and *Ecklonia maxima* ‘Kelpak’)	Foliar	3 mL L^−1^	Spray treatments at weekly intervals, starting from 17 days after sowing.	On average, both biostimulants increased yields by 48.3%.	Rouphael et al., 2018
	Plastic tunnel	Protein Hydrolysate ‘Trainer’	Foliar	4 mL L^−1^	Spray treatments at six-day intervals, starting at 21 days after sowing.	Across crop cycles and nitrogen fertilizer applications, treatments increased yields by 23.5% and improved nitrogen use and uptake efficiency.	Di Mola et al., 2020
**Rocket**	Greenhouse	Protein Hydrolysate ‘Trainer’	Foliar	3 mL L^−1^	Spray treatments at weekly intervals, from leaf length above 6 cm.	Across two successive crop cycles treatments increase yield by 11.4%	Caruso et al., 2019
**Product Quality Modulation**							
**Leafy vegetable**	**Growing Conditions**	**Biostimulant Substance**	**Application Method**	**Dosage**	**Intervention Time**	**Effect of Biostimulant Substance**	**References**
**Baby Lettuce**	Plastic tunnel	Seaweed extract ‘Kelpak’ (*Ecklonia maxima*)	Foliar	3 mL L^−1^	Spray treatments at weekly intervals, from three weeks after sowing.	33.6% Leaf ascorbic acid increase in non-fertilized plots.	Di Mola et al., 2019
	Plastic Tunnel	Protein Hydrolysate ‘Trainer’	Foliar	3 mL L^−1^	Spray treatments at weekly intervals, from three weeks after sowing.	Higher leaf succulence and leaf carotenoid contents. Antioxidant activity was nitrogen fertilization dependent.	Di Mola et al., 2019
**Lettuce**	Greenhouse	Protein Hydrolysate ‘Trainer’	Nutrient solution, foliar, and combined	0.15 and 0.3 mL ‘Trainer’ L^−1^ nutrient solution applications, foliar at 3 mL L^−1^	Foliar treatments after transplanting, and every 6 days. Nutrient solution application when refilled.	Foliar applications only increased total ascorbic acid in green butterhead by 51.2%. Combined applications increased red crisphead hydrophilic antioxidant activity by 21.9%, and total ascorbic acid 5.6-fold.	Cristofano et al., 2021
	Open Field	Humic acid	Foliar	2 and 4 mL L^−1^	Spray treatments at 4 and 6 weeks after transplanting.	The 4 mL L^−1^ treatments increased leaf phosphorous, potassium zinc, and magnesium contents. Leaf nitrate content was decreased.	Fouad Fawzy, 2010
**Spinach**	Greenhouse	Seaweed extract (*Ascophyllum nodosum*-based ‘Amalgerol’ and *Ecklonia maxima* ‘Kelpak’)	Foliar	3 mL L^−1^	Spray treatments at weekly intervals, starting from 17 days after sowing.	Increased leaf potassium and magnesium contents by 25.6 and 20.1%, increased phenolic and ascorbic acid contents by 30.7 and 79.1%. Leaf nitrate levels only 4.3% below EU limit.	Rouphael et al., 2018
	Open Field	Humic acid	Foliar	10% solution at 160 L ha^−1^	40, 50, and 60 days after germination.	Phenolic and carotenoid increases were deemed significant after the second and third treatment.	Aslam et al., 2016
**Rocket**	Greenhouse	Protein Hydrolysate ‘Trainer’	Foliar	3 mL L^−1^	Spray treatments at weekly intervals, from leaf length above 6 cm.	Averaged across two crop cycles, higher phosphorous, calcium, polyphenolic contents. Leaf Vitamin C and antioxidant activity were also recorded.	Caruso et al., 2019

**Table 3:** An overview of the abiotic stress amelioration, growth improvement, and fruit quality enhancement by biostimulant substances on Solanaceous vegetable crops.

Abiotic Stress Amelioration							
Solanaceous Crop	Growing Conditions	Biostimulant Substance	Application Method	Dosage	Intervention Time	Effect of Biostimulant Substance	References
**Tomato**	Laboratory	Silicon as silicon Nanopowder	Priming	0.5, 1, 2, and 3 mM		0.5 mM silicon rolled back germination rates of 150 mM NaCl stressed seeds up to control (0 NaCl) levels.	Almutairi, 2016
	Growth Chamber	Silicon as calcium silicate	Nutrient solution	CaSiO_3_ at the rate of 2 and 4 mM		Silicon amelioration of salt (100 mM) stress was found to be cultivar and dosage-dependent.	Wasti et al., 2017
	Greenhouse	Seaweed Extracts ‘Rygex’ and ‘Super Fifty’ (*Ascophyllum nodosum*)	Substrate Drench	0.25 and 0.20% solution for ‘Rygex’ and ‘Super Fifty’	Treatments were applied every two weeks	‘Super Fifty’ increased plant fresh weight by 6%, no yield increase. ‘Rygex’ decreased fruit fresh weight by 17.1%.	Di Stasio et al., 2018
	Greenhouse	Seaweed Extract ‘Bio-algeen S92’ (*Ascophyllum nodosum*)	Foliar Spray	0.20%	Two treatments were applied immediately after transplanting, and fifteen days later.	Compared to drought stressed plants, treated plants had higher plant growth and fruit yield	Murtic et al., 2018
	Growth Chamber	Protein Hydrolysate ‘Trainer’	Foliar Spray	3 mL L^−1^	Two treatments were applied at 8 and 15 days after planting	Compared to iron deficiency-stressed plants, treatments reduced iron reductase activity and increased shoot iron contents.	Celletti et al., 2020
	Open Field	Protein Hydrolysate ‘CycloFlow’	Soil applications	3 g L^−1^	Treatments were applied at transplanting, and every 15 days	Compared to drought stressed plants, treatments increased pollen viability. Plant yield increased six-fold.	Francesca et al., 2021
	Greenhouse	Humic Acid	Soil applications	750 and 1500 mg L^−1^	Treatments were applied at 10, 25, and 40 days after transplanting	750 mg L^−1^ treatments increased fruit yield by 27.5% compared to salt-stressed plants.	Feleafel and Mirdad, 2014
**Pepper**	Growth Chamber	Silicon as potassium silicate	Nutrient Solution	2 mM K_2_SiO_3_		Silicon increased dry weights, leaf area, and photosynthesis, the effect was cultivar dependent.	Altuntas et al., 2018
	Greenhouse	Seaweed Extract (*Ascophyllum nodosum*)	Soil Drench	1, 2, and 3 g L^−1^	Treatments were applied every week with irrigation	When compared to 100 mM NaCl salt-stressed controls, treated plant showed higher yield and lower stress related parameters.	Yildiztekin et al., 2018
**Hot Pepper**	Greenhouse	Humic Acid	Substrate incorporation	750 and 1500 mg L^−1^ and combined HA and calcium nitrate		750 mg L^−1^ treatments, alone and combined with calcium increased growth and yield parameters compared to 100 mM salt stressed controls.	Akladious and Mohamed, 2018
**Plant Growth and Yield Enhancement**							
**Solanaceous Crop**	**Growing Conditions**	**Biostimulant Substance**	**Application Method**	**Dosage**	**Intervention Time**	**Effect of Biostimulant Substance**	**References**
**Tomato**	Greenhouse	Seaweed extract (*Chaetomorpha antennina*)	Seed Priming	Seaweed extract at concentration from 20 to 100%		100% Extract increased plant growth. Tomato yield was increased by 135.9%	Muthu-Pandian Chanthini et al., 2019
	Field and Greenhouse	Seaweed extract (*Ascophyllum nodosum*)	Foliar spray and soil drench	0.2 and 0.5%	Treatments were administered 15 days after transplanting, and every 15 days thereafter.	0.5% spray treatment was the most effective, increasing yield by 63% in field, and 54% in greenhouse.	Ali et al., 2016
	Open Field	Protein Hydrolysate ‘Trainer’	Foliar Spray	3 mL L^−1^	Weekly spray intervals, starting from the early growth of the first fruit truss.	Treated plants recorded 18.6% higher yields due to 19.3% higher fruit numbers.	Caruso et al., 2019
	Greenhouse	Protein Hydrolysate ‘Trainer’	Foliar spray	2.5 and 5 mL L^−1^	Spray treatments at 10-day intervals, starting from 15 days after transplanting.	5 mL L^−1^ differentially increased yields in both tested tomato cultivars. ‘Akyra’ recorded 13.9% higher fruit numbers, ‘Sir Elyan’ 28.7 heavier than their respective controls.	Rouphael et al., 2017
	Greenhouse	Humic substance ‘Humicop’	Substrate incorporation	100 L ha^−1^		Treated plants recorded increased yields by 18.1%.	Abou Chehade et al., 2018
	Open Field	Humic Acid	Soil incorporation	40–80–120–160–200 L ha^−1^		160 and 200 L ha^−1^ treatments increased yields by 35.2% and leaf nutrition.	Asri et al., 2015
**Pepper**	Greenhouse	Seaweed extract ‘Wokozim’ (*Ascophyllum nodosum*)	Foliar spray	2 and 4 mL L^−1^	Spray treatments at 15 day intervals.	4 mL L^−1^ sprays increased yields by 83 and 46.4% in cultivars ‘Sven Rz’ and ‘Red Knight’.	Khan et al., 2018
	Greenhouse	Humic substance ‘Solum H80’	Foliar Spray	0.5, 1, and 1.5 g L^−1^	Spray treatments at 20-day intervals, starting from 20 days after transplanting.	1.5 g L^−1^ elicited 15.7, 7.2, and 14.1% higher yields from the three tested cultivars due to a modulation of yield parameters.	Ibrahim et al., 2019
**Eggplant**	Open Field	Seaweed extract ‘Göemar BM-86’ (*Ascophyllum nodosum*)	Foliar spray	1.5 L biostimulant ha^−1^	Spray treatments every two weeks, staring from 2 weeks after transplanting.	Of the 6 tested cultivars, ‘Epic’, ‘Flavine’, and ‘Wa 6020 F10’ registered yield increases across two growing seasons.	Pohl et al., 2019
**Fruit quality modulation**							
**Solanaceous Crop**	**Growing Conditions**	**Biostimulant Substance**	**Application Method**	**Dosage**	**Intervention Time**	**Effect of Biostimulant Substance**	**References**
**Tomato**	Greenhouse	Seaweed extract (*Chaetomorpha antennina*)	Seed Priming	Seaweed extract at concentration from 20 to 100%		Increased total soluble solids (+8.5%), phenolics (+74.6%), and ascorbic acid contents (+38.9%).	Muthu-Pandian Chanthini et al., 2019
	Greenhouse	Seaweed Extract ‘Bio-algeen S92’ (*Ascophyllum nodosum*)	Foliar Spray	0.20%	Two treatments were applied immediately after transplanting, and fifteen days later.	Increased total soluble solids (+3.1%), phenolics (+10.8%), and FRAP antioxidant activity (10.2%).	Murtic et al., 2018
	Greenhouse	Seaweed extract ‘Kelpak’ (*Ecklonia maxima*)	Foliar Spray	3 mL L^−1^	10-day spray intervals, starting from the early growth of the first fruit truss.	No increase in total soluble solids, juice pH, antioxidant activity, total phenol contents, ascorbic acid, lycopene.	Colla et al., 2017
	Greenhouse	Protein Hydrolysate ‘Trainer’	Foliar spray	2.5 and 5 mL L^−1^	Spray treatments at 10-day intervals, starting from 15 days after transplanting.	5 mL treatment performed best, by increasing fruit total soluble solid, +10.7%; lipophilic, +260%; and hydrophilic, +61.9% antioxidant activity. Lycopene increased by 34.9%.	Rouphael et al., 2017
	Open Field	Protein Hydrolysate ‘Trainer’	Foliar Spray	3 mL L^−1^	Weekly spray intervals, starting from the early growth of the first fruit truss.	Treatments increased fruit total soluble solids, 10.1%; lipophilic antioxidants, 56.9%; lycopene, 30.7%; and ascorbic acid, 106.2%	Caruso et al., 2019
	Open Field	Humic Acid	Soil incorporation	40–80–120–160–200 L ha^−1^		No increase in TSS across two growing seasons. Titratable acidity increase across two growing seasons was 10.3%	Asri et al., 2015
**Pepper**	Greenhouse	Humic substance ‘Solum H80’	Foliar Spray	0.5, 1, and 1.5 g L^−1^	Spray treatments at 20-day intervals, starting from 20 days after transplanting.	The 1.5 g L^−1^ treatment was the most performing, by increasing ascorbic acid content, titratable acidity, total soluble solids, and total sugar. Increase was cultivar-dependent.	Ibrahim et al., 2019

## Data Availability

Not applicable.

## Abbreviations

ABA	Abscisic acid
AMF	Arbuscular mychorrhizal fungi
ANE	*Ascophyllum nodosum* extract
APX	Ascorbate peroxidase
AQP	Aquaporin
BL	Brassinolide
BS	Biostimulant Substance
CAT	Catalase
CK	Cytokinins
CS	Castasterone
FA	Fulvic Acid
FRAP	Ferric reducing antioxidant activity
GA	Gibberellic acid
GPX	Glutathione peroxidase
HA	Humic Acid
HS	Humic Substance
IAA	Indol-3-acetic acid
LAI	Leaf area index
MDA	Malondialdehyde
NIP	Nodulin 26-like intrinsic protein
PGP	Plant growth promoting
PGR	Plant growth regulator
PH	Protein Hydrolysate
POD	Peroxydase
ROS	Reactive Oxygen Species
SOD	Superoxide dismutase
SOS	Sodium overly sensitive
SPAD	Soil plant analysis development
SWE	Seaweed Extract
TCA	Tricarboxylic acid cycle
